# Supplementary material for: Next-Generation Sequencing of the Human Aqueous Humour Microbiome
Source: Int J Mol Sci. 2024 Jun 1;25(11):6128. doi: 10.3390/ijms25116128 (PMC11173048; doi:10.3390/ijms25116128)
Supplement: Supplementary file 1 [file ijms-25-06128-s001.zip › ijms-3019174-supplementary.pdf]

International Journal of *Molecular Sciences*

MDPI

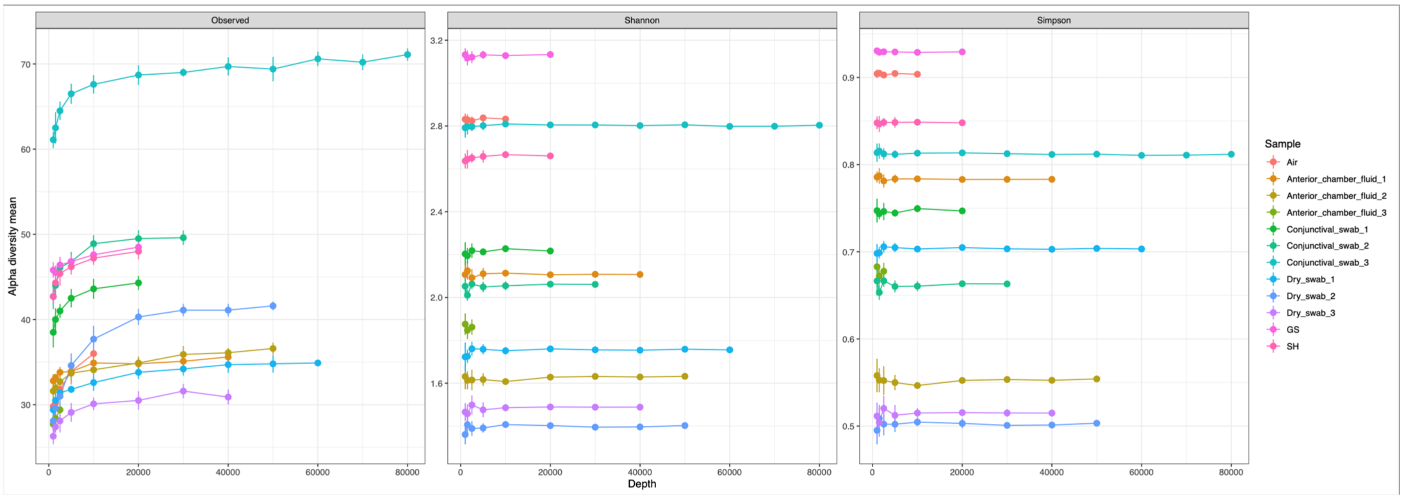

Figure S1. Observed Amplicon Sequence variant, Simpson and Shannon index displaying alpha diversity of the different samples.

unifrac	Air	ACF_1	ACF_2	ACF_3	CS_1	CS_2	CS_3	DS_1	DS_2	DS_3	GS	SH
Air	0	0.747	0.670	0.630	0.582	0.657	0.662	0.532	0.734	0.715	0.493	0.596
ACF_1	0.747	0	0.460	0.428	0.414	0.454	0.407	0.434	0.425	0.440	0.816	0.867
ACF_2	0.670	0.460	0	0.167	0.245	0.162	0.351	0.349	0.109	0.0841	0.684	0.737
ACF_3	0.630	0.428	0.167	0	0.176	0.172	0.314	0.306	0.219	0.204	0.586	0.636
CS_1	0.582	0.414	0.245	0.176	0	0.235	0.278	0.274	0.272	0.281	0.552	0.631
CS_2	0.657	0.454	0.162	0.172	0.235	0	0.228	0.334	0.213	0.192	0.656	0.693
CS_3	0.662	0.407	0.351	0.314	0.278	0.228	0	0.341	0.315	0.337	0.663	0.724
DS_1	0.532	0.434	0.349	0.306	0.274	0.334	0.341	0	0.341	0.328	0.708	0.790
DS_2	0.734	0.425	0.109	0.219	0.272	0.213	0.315	0.341	0	0.0733	0.759	0.812
DS_3	0.715	0.440	0.0841	0.204	0.281	0.192	0.337	0.328	0.0733	0	0.735	0.793
GS	0.493	0.816	0.684	0.586	0.552	0.656	0.663	0.708	0.759	0.735	0	0.325
SH	0.596	0.867	0.737	0.636	0.631	0.693	0.724	0.790	0.812	0.793	0.325	0

Figure S2. Weighted Unifrac results representing the basis for the display of the beta diversity of the samples (displayed in Figure2). Abbreviation: ACF (anterior chamber fluid), CS (conjunctival swab), DS (dry swab).
